# *Clostridium difficile*–associated Disease in the Elderly, United States 

**DOI:** 10.3201/eid1502.080785

**Published:** 2009-02

**Authors:** Jyotsna Jagai, Elena Naumova

**Affiliations:** Tufts University School of Medicine, Boston, Massachusetts, USA

**Keywords:** Clostridium difficile, hospitalizations, seasonality, elderly, United States, letter

**To the Editor:** Zilberberg et al. (*1*) recently commented on the increase of hospitalizations for *Clostridium difficile*–associated disease (CDAD) and noted an increase in the case-fatality rate during 2000–2005. These findings refer to the entire US adult population and agree with our observations for the elderly (>65 years of age). We assessed trends of CDAD in the elderly by using hospital billing data from the Centers for Medicare and Medicaid Services (CMS), which covers 98% of the elderly population (*2*). We abstracted all 1,054,125 hospitalization records that included *C*. *difficile* (International Classification of Diseases, 9th revision, Clinical Modification [ICD 9-CM], diagnosis code 008.45) in any of the 10 diagnosis code positions for a 14-year period (1991–2004). We used elderly-population data from the 1990 and 2000 US Census. The ICD code for *C*. *difficile* was introduced in 1992. Case-patients in our dataset prior to this date represent severe illness and were hospitalized for >1 year and therefore were still in the hospital when the ICD code was introduced. We considered data from 1993 through 2004 because 1991 and 1992 are not representative due to introduction of the ICD code.

We observed an increase in overall hospitalizations that included a diagnosis for CDAD (Appendix Figure) and an increase in rates of CDAD from 13.71/10,000 elderly in 1993 to 38.78/10,000 in 2004 (*3*). The highest rate of hospitalizations was detected in the oldest patients (>85 years of age), 48.2/10,000 vs. 11.9 in those 65–74 years of age and 26.0 in those 75–84 years of age (*3*). These rates might be higher than rates reported by Zilberberg et al. because our records account for all treated conditions recorded by all 10 diagnosis codes. The ICD code for CDAD typically does not appear in the primary and secondary diagnosis; overall, 60% of all CMS records list CDAD as codes 3–10 (*3*). Primary and secondary codes typically represent diagnoses for which the patient is admitted, whereas diagnosis codes 3–10 are codes used for chronic conditions and sequelae. The Appendix Figure, panel A, shows the change in the proportion of CDAD cases in each diagnosis code over the study period. The proportion of CDAD in the primary and secondary diagnosis position increased during 1996–1997; however, this proportion is stabilizing at ≈25%.

Zilberberg et al. observed a doubling in age-adjusted case-fatality rates from 1.2% in 2000 to 2.2% in 2004 (*1*), which is an annual increase of 0.2% over the 5-year period. We are not able to calculate case-fatality rate by using CMS data because these data do not provide cause of death, only an indicator of whether the patient died during that hospital stay. However, we observed an increase in the percentage of patients with CDAD who died, from 8.8% in 1993 to 9.7% in 2004, which is an annual increase of 0.075% over the 12-year period. We also observed a peak in 2000; 10.4% of patients with CDAD died. This peak is unusual and unexplained and requires further analysis. Data on deaths must be interpreted with caution because they may be affected by severe conditions and age (oldest patients).

We observed an increasing trend and strong seasonal pattern in CDAD hospitalizations. The Appendix Figure, panel B, shows this seasonal pattern by week during 1993–2004. This figure shows an increasing trend over time with a sharp change in slope in 2001. This increasing trend may represent an increase in disease or may be caused by increased testing and recognition of disease. Diagnosis of CDAD in the United States is now made by using an enzyme immunoassay that is relatively easier and cheaper to perform than a cytotoxin assay (*4*), which may account for the increased trend.

Increases in rates of CDAD may be caused by a reporting bias of gastroenteric diseases (*5*–*7*). To assess this possibility, we extracted all records that included other infectious gastroenteritis without CDAD (all other gastrointestinal [GI] infections, ICD 001–009 without 008.45) and compared the trend with CDAD hospitalizations (Appendix Figure, panel B). The Appendix Figure shows that rates for all other GI infections remained fairly constant over the study period, and a reporting bias for GI infections does not account for the ≈3-fold increase in CDAD hospitalizations. CDAD hospitalization rates for the elderly also show a strong annual seasonal pattern (Appendix Figure, panel B), which was estimated to peak in the second week of March, the 10th week of the year. This seasonality suggests dominant routes of transmission that may be environmentally driven.

Our findings support the observations of Zilberberg et al. and demonstrate the substantial increase in CDAD-related hospitalizations over time. These findings and the aging population in the United States underscore the need for further research to understand all aspects of CDAD.

## Supplementary Material

 Appendix Figure Trends in hospitalization cases for Clostridium difficile-associated disease (CDAD) in the elderly (>65 years of age) in the United States over a 12-year period (1993-2004). A) Changes in position of CDAD diagnosis code in hospitalization records over the period. The black line in panel A shows increasing number of hospitalizations (in thousands of cases) for CDAD over the same period. B) Weekly rate (per 10,000 elderly) of hospitalizations for CDAD (black dots) compared with hospitalizations for all other gastrointestinal (GI) infections (red dots). Solid lines represent smoothed seasonal patterns.
