# Supplementary figures and images for: Clostridium difficile–associated Disease in the Elderly, United States
Source: Emerg Infect Dis. 2009 Feb;15(2):343–4. doi: 10.3201/eid1502.080785 (PMC2662653; doi:10.3201/eid1502.080785)

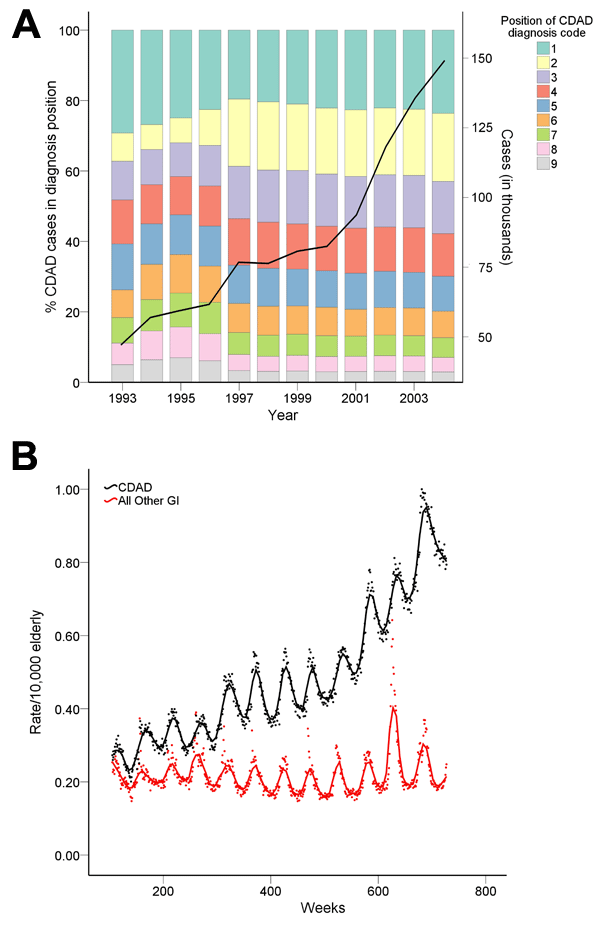

Supplement: Appendix Figure — Trends in hospitalization cases for Clostridium difficile-associated disease (CDAD) in the elderly (>65 years of age) in the United States over a 12-year period (1993-2004). A) Changes in position of CDAD diagnosis code in hospitalization records over the period. The black line in panel A shows increasing number of hospitalizations (in thousands of cases) for CDAD over the same period. B) Weekly rate (per 10,000 elderly) of hospitalizations for CDAD (black dots) compared with hospitalizations for all other gastrointestinal (GI) infections (red dots). Solid lines represent smoothed seasonal patterns. [file 08-0785_app-s1.gif]
